# Tobacco Use and Nicotine Dependence among Conflict-Affected Men in the Republic of Georgia

**DOI:** 10.3390/ijerph10062185

**Published:** 2013-05-29

**Authors:** Bayard Roberts, Ivdity Chikovani, Nino Makhashvili, Vikram Patel, Martin McKee

**Affiliations:** 1 European Centre on Health of Societies in Transition, London School of Hygiene and Tropical Medicine, 15-17 Tavistock Place, London WC1H 9SH, UK; E-Mail: martin.mckee@lshtm.ac.uk; 2 Curatio International Foundations, Tbilisi 0162, Georgia; E-Mail: i.chikovani@curatio.com; 3 Global Initiative on Psychiatry, Tbilisi 0162, Georgia; E-Mail: nino.makhashvili.1@iliauni.edu.ge; 4 Ilia State University, Tbilisi 0162, Georgia; 5 Centre for Global Mental Health, London School of Hygiene and Tropical Medicine, 15-17 Tavistock Place, London WC1H 9SH, UK; E-Mail: vikram.patel@lshtm.ac.uk

**Keywords:** tobacco, nicotine, Georgia, war, forced displacement

## Abstract

*Background*: There is very little evidence globally on tobacco use and nicotine dependence among civilian populations affected by armed conflict, despite key vulnerability factors related to elevated mental disorders and socio-economic stressors. The study aim was to describe patterns of smoking and nicotine dependence among conflict-affected civilian men in the Republic of Georgia and associations with mental disorders. *Methods*: A cross-sectional household survey using multistage random sampling was conducted in late 2011 among conflict-affected populations in Georgia. Respondents included in this paper were 1,248 men aged ≥18 years who were internally displaced persons (IDPs) and former IDPs who had returned in their home areas. Outcomes of current tobacco use, heavy use (≥20 cigarettes per day), and nicotine dependence (using the Fagerström Test for Nicotine Dependence) were used. PTSD, depression, anxiety and hazardous alcohol use were also measured, along with exposure to traumatic events and a range of demographic and socio-economic characteristics. *Results*: Of 1,248 men, 592 (47.4%) smoked and 70.9% of current smokers were heavy smokers. The mean nicotine dependence score was 5.0 and the proportion with high nicotine dependence (≥6) was 41.4%. In multivariate regression analyses, nicotine dependence was significantly associated with PTSD (β 0.74) and depression (β 0.85), along with older age (except 65+ years), and being a returnee (compared to IDPs). *Conclusions*: The study reveals very high levels of heavy smoking and nicotine dependence among conflict-affected persons in Georgia. The associations between nicotine dependence, PTSD and depression suggest interventions could yield synergistic benefits.

## 1. Background

There are currently an estimated 40 million persons that have been forcibly displaced from their home areas by armed conflict, the vast majority of whom live in low and middle income countries. They include over 26 million internally displaced persons (IDPs) who remain within the borders of their countries and up to 14 million refugees and stateless persons who are living in other countries [1,2]. There are millions more civilians who remain entrapped in conflict-affected areas or in places that were, until recently, beset by conflict. Many conflict-affected persons will have been exposed to violent and traumatic events, placing them at high risk of post-traumatic stress disorder (PTSD) [3]. Many conflict-affected populations also experience an increased burden of common mental disorders, particularly depression and anxiety, due to a combination of trauma exposure, poor living conditions, loss of livelihoods, impoverishment, and other stressors [3,4,5].

As research in populations free from conflict has shown that greater tobacco use and nicotine dependence are associated with both post-traumatic stress disorder (PTSD) and common mental disorders such as depression, anxiety and alcohol use [6,7,8,9,10,11,12], it might be expected that those exposed to conflict may be more vulnerable to heightened tobacco use. Indeed, there is increasing concern over chronic diseases among conflict-affected populations [13], including tobacco-related diseases. Yet despite this potential vulnerability for heightened tobacco use, there are very few studies on tobacco use among conflict-affected civilian populations and they are generally characterised by small sample sizes and limited analysis, with only a few examining associations between tobacco use and mental disorders or trauma exposure. Identified papers include those addressing: smoking patterns of 989 Kurdish youth in Iraq [14]; antismoking messages and current cigarette smoking status among 1,122 youth in Somaliland [15]; current smoking and smoking cessation rates among 740 elderly people (including refugees) in Beirut, Lebanon [16]; variances in smoking rates between 32 adolescent IDPs with 528 non-IDPs adolescents in Belgrade, Serbia [17]; links between subjective threat of armed conflict and psychosocial outcomes (including cigarette smoking) among 24,935 conflict-affected Israeli and Palestinian youth [18]; tobacco use among 194 immigrant and refugee youth in British Columbia, Canada [19]; and smoking patterns, nicotine dependence and correlations with PTSD among 66 Bosnian refugees in a primary care setting in the United States [20].

Better understanding of patterns and determinants of smoking is a first step in strengthening tobacco control, tackling tobacco use and its effects and thus of improving the long-term health of conflict-affected populations. This paper takes advantage of data collected as part of a broader study on mental health among conflict-affected populations in Georgia to examine smoking and nicotine dependence among conflict-affected civilian men in the Republic of Georgia.

### The Republic of Georgia

The Republic of Georgia has experienced two main phases of conflict. The first, in the early 1990s, involved secessionist movements in the regions of Abkhazia and South Ossetia, with fighting leading to displacement of approximately 300,000 people, of whom approximately 200,000 have not returned to their home areas. The second was in August 2008, when conflict broke out between Georgia and the Russian Federation over South Ossetia, leading to at least 128,000 ethnic Georgians being displaced, of which up to 100,000 have returned to their home areas in the border region with South Ossetia [21]. The majority of current IDPs live in government-established IDP settlements while some remain in makeshift settlements in former hotels, schools, factories, and hospitals. IDP communities are commonly characterised by poor living conditions, high unemployment, poverty, limited integration with local communities, and low access to health care [21,22]. The living conditions and economic prospects of many who have returned to the border region with South Ossetia are also poor, with high unemployment and vulnerability to future conflict arising from their proximity to the border. To the best of our knowledge, no studies have been conducted on smoking among conflict-affected Georgians. 

The aim of this paper is to describe patterns of smoking and nicotine dependence among conflict-affected civilian men in the Republic of Georgia and associations with mental disorders. The specific objectives are to: (i) measure the prevalence of current and heavy smoking; (ii) describe the pattern of nicotine dependence; and (iii) examine the influence of PTSD and common mental disorders with nicotine dependence. Smoking rates among women in these populations are very low (see below) so the analyses were restricted to men. 

## 2. Methods

The study uses a cross-sectional survey design and multi-stage random sampling, with stratification by region and displacement status, which was representative of the conflict-affected populations in Georgia. A total sample size of 3,600 was determined to meet the statistical requirements of the overall study. This consisted of 1,200 respondents from each of the three main conflict-affected populations groups in Georgia: those displaced as a result of conflicts in the 1990s (“old IDPs”), those displaced after the 2008 conflict (“new IDPs”) and 1,200 former “new IDPs” who have returned to their home areas (“returnees”).

Primary sampling units (N = 360; 120 per population group) were selected based on probability proportion to size method using a sampling frame of a list of old and new IDP settlement population sizes provided by the Ministry of Internally Displaced Persons, and lists of villages in the border region with South Ossetia provided by Shida Kartli region Governor’s office. These were considered to be the most accurate list available. Within each primary sampling unit, the random walk method was used to select households, with a starting address is randomly selected and, taking alternate left- and right-hand turns at road junctions, every nth address is selected [23]. The same number of households per cluster was selected to maintain the sample weighting generated through the probability to proportion to size method. Within the selected household one person (aged ≥ 18 years) was selected to be interviewed (based on nearest birthday). If there was no response at the household after 3 visits (on different days and at different times), the next household on the route was visited (using the random walk method).

Trained fieldworkers conducted face-to-face interviews in the respondents’ homes. All interviews were conducted in Georgian. The response rate was 79%. Replacement respondents were randomly sampled from the same primary sampling units to ensure the desired sample of 3,600 respondents. Data collection took place between October and December 2011. Full respondent anonymity was assured. All respondents provided informed consent prior to their inclusion in the study. Ethics approval was provided by the National Council on Bioethics in Georgia and the London School of Hygiene and Tropical Medicine.

Current smoking was defined as currently smoking at least one cigarette, papirossi (filterless cigarette), pipe, cigar daily. The outcome of nicotine dependence was measured using the Fagerström Test for Nicotine Dependence (FTND) which has been validated and widely used [24]. FTND consists of six items with scores attached to the response options. The sum of these items is then produced, with higher FTND scores indicating a more intense physical dependence on nicotine. For descriptive analyses, scores were also allocated to categories, as follows: low dependence (FTND score of <4), moderate dependence (FTND score of 4–5), and high nicotine dependence (FTND score of ≥6) [24].

PTSD was measured using the Trauma Screening Questionnaire (TSQ) which consists of 10 items on PTSD symptoms over the past 1 week, with No (= 0) and Yes (= 1) responses which are summed to produce an overall score range of 0–10, with TSQ’s cut-off of ≥6 used to indicate possible PTSD [25]. Three common mental disorders of depression, anxiety and alcohol disorder were used in this study. Depression was measured using the Patient Health Questionnaire (PHQ-9) which consists of 9 questions on depression symptoms over the last 2 weeks, with item scores summed to produce a total score range of 0–27, with the PHQ-9’s suggested cut-off of ≥10 used to indicate at least moderate depression [26]. Anxiety was measured using the Generalised Anxiety Disorder (GAD-7) instrument which consists of 7 questions on anxiety symptoms over the last 2 weeks, which produces a total score range of 0–21, with the GAD-7’s suggested cut-off of ≥10 used to indicate at least moderate anxiety [27]. TSQ, PHQ-9, and GAD-7 showed good reliability with Cronbach’s alpha scores of 0.86, 0.86, 0.90 respectively; and results from a separate test-retest mini survey (N = 110) showed intraclass correlation (ICC) results 0.97, 0.98, and 0.96 respectively. Alcohol disorder was measured using the Alcohol Use Disorders Identification Test (AUDIT) which consists of 10 items with a recall period of the previous 1 year, with AUDITs suggested cut-off of ≥8 used to indicate hazardous alcohol use [28]. Lifetime exposure to violent and traumatic events was also assessed, using an adapted version of the Harvard Trauma Questionnaire (see Table 1 for items) which were treated as both individual items and also cumulatively (0, 1, 2, >3 events) [29]. The questionnaire also included a range of demographic and socio-economic items.

### Analysis

The analysis was limited to men only, as only 30 (1.17%) of the 2,352 women reported being daily smokers which prevented any meaningful analysis. This low number of women smokers reflects the low smoking levels among women in Georgia [30,31], particularly from rural areas (which is where the majority of conflict-affected women come from), but it may also reflect an under-reporting related to stigma in rural Georgia associated with women smoking. There is no such stigma for Georgian men reporting on their smoking behaviour. Nine percent of current male smokers did not complete all the FTND items and so they were omitted from the FTND analysis. There was no statistically significant difference between the characteristics of completed participants and non-completed participants to suggest any bias associated with their exclusion.

**Table 1:** Sample characteristics (men only, N = 1,248).

	N	(%)
**Age:**		
18–39 years	458	(36.69)
40–59 years	441	(35.31)
60+ years	349	(28.00)
**Marital status:**		
Married/cohabiting	816	(65.37)
Single	347	(27.82)
Widowed	85	(6.81)
**Education status:**		
Completed higher education	253	(20.27)
Completed secondary school	872	(69.87)
Primary/incomplete secondary	123	(9.86)
**Household economic status:**		
Very good	7	(0.59)
Good	23	(1.86)
Average	551	(44.19)
Bad	460	(36.85)
Very bad	206	(16.50)
**Trauma exposure:**		
Lack of shelter	651	(52.17)
Serious injury	309	(24.74)
Directly in combat situation	635	(50.87)
Physical abuse	29	(2.30)
Been tortured	36	(2.85)
Witnessed murder, violence acts against family/friends	310	(24.81)
Witnessed murder, violence acts against stranger	129	(10.36)
Death of family member/close friend during conflict	385	(30.88)
**Mental disorders:**		
PTSD ^1^	235	(19.16)
Depression ^2^	142	(11.40)
Anxiety ^3^	96	(7.66)
Hazardous alcohol use ^4^	232	(25.75)

^1^ Score of >5 on the TSQ instrument. ^2^ Score of ≥10 on the PHQ-9 instrument. ^3^ Score of ≥10 on the GAD-7 instrument. ^4^ Score of ≥8 on the AUDIT instrument.

Correlations with nicotine dependence of the mental disorders and also trauma exposure were examined using linear regression analyses (as the FTND produces a continuous outcome variable). Given the known socio-economic risk-factors for nicotine dependence [32,33], regression analysis was also run for variables for age, educational attainment, marital status, household economic status, employment status, general living conditions and displacement status (old and new IDPs were combined in the final analysis as preliminary analysis showed no significant differences in smoking levels or nicotine dependence between these two groups). The variables which showed a significant association (*p* < 0.05) with increasing dependence in bivariate analysis were then entered into a multivariate model in order to adjust for the influence of the other included variables.

The data were weighted to reflect the actual proportions of “old IDPs”, “new IDPs” and “returnees” in the overall conflict-affected population of Georgia, based upon the sampling frames noted above. Data were also adjusted for the cluster survey design. Statistical significance was assumed at *p* < 0.05.

## 3. Results

In total, 3,600 respondents were interviewed. Of these, 1,248 (34.7%) were men. This reflects findings of other studies of the general population in Georgia as many men have left to find employment elsewhere [34]. The mean age of the men was 47.7 years (95% CI 46.7; 48.7). 235 men (19.2%) were recorded with PTSD, 142 (11.4%) with depression, 96 (7.7%) with anxiety, and 236 (26.2%) with hazardous alcohol use. Other sample characteristics are shown in Table 1.

**Figure 1:**
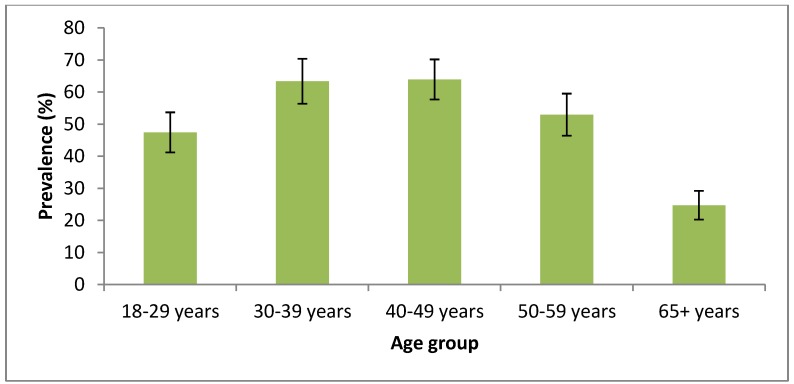
Prevalence of current smoking (with 95% error bars), by age group (men only, N = 1,248).

Of the total 1,248 men, 592 (47.4% (95% CI 44.65; 50.20)) were current smokers. The levels of current smoking by age group are shown in Figure 1, with the highest rates among men aged 30–39 years (63.4%) and 40–49 years (63.9%). The prevalence of current smoking among male IDPs was 51.2% (95% CI 47.71; 54.58), while for male returnees it was 40.4% (95% CI 35.72; 45.02). Of all the current male smokers, 70.9% (95% CI 67.17; 74.68) smoked ≥20 cigarettes per day (68.9% (95% CI 64.25; 73.47) among IDPs; 75.9% (95% CI 69.44; 82.28) among returnees).

The mean FTND score for male smokers was 5.0 (95% CI 4.81; 5.19) and the proportion with high nicotine dependence (≥6) was 41.4% (95% CI 37.26; 45.62) (Table 2). Despite a higher prevalence of smoking among IDPs, the mean nicotine dependence score was slightly higher (though not significantly so) among returnees (5.38 (95% CI 5.05; 5.71)) than IDPs (4.83 (95% CI 4.60; 5.07)). There was a similar pattern for high nicotine dependence, with a prevalence among returnees of 47.0% (95% CI 39.40; 54.65) compared to 39.0% (95% CI 34.02; 44.03) among IDPs.

**Table 2:** Results of individual FTND items and the mean and categorical FTND scores (male current smokers only, N = 565).

	N	%	(95% CI)	
**Individual FTND items**				
*Number of cigarettes:*				
1 to 10	130	(22.92)	(19.45;	26.40)
11 to 20	313	(55.45)	(51.34;	59.56)
21 to 30	47	(8.39)	(6.09;	10.68)
Over 30	75	(13.24)	(10.44;	16.04)
*How soon smoke first cigarette after waking up:*				
After 60 min	24	(4.32)	(2.63;	6.01)
30–60 min	102	(18.20)	(14.99;	21.41)
5–30 min	182	(32.63)	(28.73;	36.53)
<5 min	251	(44.85)	(40.71;	48.98)
*Difficulty in smoking in places where not allowed:*				
No	354	(64.11)	(60.10;	68.13)
Yes	198	(35.89)	(31.87;	39.90)
*Smoke more during first hours after waking than in rest of the day:*				
No	281	(50.84)	(46.66;	55.02)
Yes	272	(49.16)	(44.98;	53.34)
*Which cigarette most unwilling to give up:*				
Any of the others	387	(68.97)	(65.13;	72.81)
First in the morning	174	(31.03)	(27.19;	34.87)
*Do you smoke even when you are very ill:*				
No	282	(50.06)	(45.92;	54.20)
Yes	281	(49.94)	(45.80;	54.08)
**Summary FTND score**				
Mean FTND score	536	(5.00)	(4.81;	5.19)
% with low dependence ^1^	134	(24.94)		
% with moderate dependence ^2^	180	(33.62)		
% with high dependence ^3^	222	(41.44)	(37.26;	45.62)

CI, confidence interval. ^1^ FTND score of <4. ^2^ FTND score of 4–5. ^3^ FTND score of ≥6. Only respondents that answered all FTND items included in summary FTND score.

In the adjusted regression models, depression (β 0.85) and PTSD (β 0.74) were significantly associated with nicotine dependence (Table 3). For PTSD, it was the hyperarousal symptoms of irritability/outbursts of anger (β 0.81, *p* < 0.01) and difficulty falling or staying asleep (β 0.58, *p* < 0.05) that were significantly associated with nicotine dependence. Neither anxiety nor hazardous alcohol use were associated with nicotine dependence. The trauma exposure variables showed no association with nicotine dependence in the multivariate analysis (neither individually nor collectively). Of the demographic and socio-economic variables, older age (except 65+ years) was associated with nicotine dependence; as was being a returnee (β 0.57) when compared to IDPs.

**Table 3:** Association of trauma exposure, PTSD, depression and alcohol consumption with nicotine dependence (men only, N = 536).

	N	Bivariate analysis				Multivariate analysis			
		β	(95% CI)		*p*	β	(95% CI)		*p*
**Age**									
18–29 years	104	Ref				Ref			
30–39 years	104	**0.95**	**(0.30;**	**1.60)**	**<0.01**	**0.90**	**(0.24;**	**1.56)**	**0.01**
40–49 years	137	**1.13**	**(0.48;**	**1.78)**	**<0.01**	**0.97**	**(0.32;**	**1.62)**	**<0.01**
50–59 years	110	**0.81**	**(0.11;**	**1.50)**	**0.02**	**0.68**	**(0.00;**	**1.36)**	**0.05**
65+ years	81	**0.77**	**(0.04;**	**1.51)**	**0.04**	0.53	(−0.21;	1.27)	0.16
**Displacement status**									
IDP	368	Ref							
Returnee	168	**0.54**	**(0.12;**	**0.97)**	**0.01**	**0.57**	**(0.14;**	**1.00)**	**0.01**
**PTSD ^1^**									
No PTSD	417	Ref				Ref			
PTSD	108	**0.79**	**(0.17;**	**1.40)**	**0.01**	**0.74**	**(0.14;**	**1.34)**	**0.02**
**Depression ^2^**									
No depression	475	Ref				Ref			
Depression	61	**0.77**	**(0.02;**	**1.52)**	**0.04**	**0.85**	**(0.11;**	**1.59)**	**0.03**
**Anxiety ^3^**									
No anxiety	484	Ref				Ref			
Anxiety	52	0.81	(−0.04;	1.66)	0.06	-			
**Hazardous alcohol use ^4^**									
No hazardous alcohol use	295	Ref				Ref			
Hazardous alcohol use	146	0.06	(−0.45;	0.57)	0.82	-			

β, beta coefficient; CI, confidence interval. ^1^ Score of >5 on the TSQ instrument. ^2^ Score of ≥10 on the PHQ-9 instrument. ^3^ Score of ≥10 on the GAD-7 instrument. ^4^ Score of ≥8 on the AUDIT instrument. - Not entered into multivariate analysis as not significant in the bivariate analysis. Separate models runs for PTSD and depression. Data in bold signify statistically significant results (*p* < 0.05).

## 4. Discussion

This study provides new evidence on the association between PTSD and common mental disorders and nicotine dependence among conflict-affected civilian populations. Its contribution to evidence on smoking among conflict-affected populations includes the use of nicotine dependence rather than just smoking, a range of mental disorders, larger sample size than most comparable studies, the study population comprised IDPs rather than refugees who have moved to high income settings, and also that it examined patterns among returned IDPs. The findings show almost half (47.4% (95% CI 44.65; 50.20)) of conflict-affected men in Georgia were current smokers. Of these current smokers, a very high proportion (70.9% (95% CI 67.17; 74.68)) could be classified as heavy smokers (≥20 cigarettes per day). A 2010 study of the general non-conflict-affected population in Georgia also recorded high levels of heavy smoking among men (65.7% (95% CI 61.16; 70.27)) [30].

The mean FTND score for men recorded in this study (5.00 (95% CI 4.81; 5.19)) is slightly, but not significantly, higher than for men in the 2010 study of the general population in Georgia (4.83 (95% CI 4.57; 5.08)) [35]. Similarly, the levels of high nicotine dependence (FTND score of ≥6) were not significantly greater among the conflict-affected men in this study (41.4% (95% CI 37.26;45.62)) compared to men in the general population in Georgia (39.6% (95% CI 33.76;45.50)) [35]. However, when compared internationally, the FTND scores are higher in our study than those recorded with general male populations in eight other countries of the former Soviet Union where the average mean score was 3.96 (ranging from 3.42 in Belarus to 4.15 in Russia) and the average level of high nicotine dependence was 25.1% (ranging from 16.7% in Belarus to 28.6% in Russia) [35].They are also higher than those recorded in Western Europe and North America [36,37,38,39]. It is not immediately clear why nicotine dependence should be so high in Georgia (both among conflict and non-conflict-affected populations) but it does reflect the high prevalence of male smoking in the country. More speculatively, smoking may be a response to the political and economic instability, insecurity and conflict experienced by Georgians over the past two decades, particularly the conflict-affected populations [40]. To compound this, there is also very limited access to smoking cessation support services in Georgia despite high levels of desire to quit smoking in the country [41].

The results indicate an association between PTSD and depression and nicotine dependence. The studies noted above of refugees relocated to Canada and the United States also report a relationship between tobacco use and poor mental health [19,20]. The study of conflict-affected Israeli and Palestinian youth suggested a link between cigarette smoking and subjective threat of armed conflict [18]. The study of 66 Bosnian refugees (39 current smokers) in a primary care setting the United States indicated an association between nicotine dependence and PTSD but it is limited by the small sample size [20]. There have been a greater number of studies on tobacco use among military combatants and war veterans and these have shown elevated levels of tobacco use and nicotine dependence and associations between PTSD, depression and nicotine dependence [10,42]. The link between poor mental health and nicotine dependence reflects study findings among non-conflict-affected populations, including the link between PTSD and nicotine dependence [7,9,43,44,45,46,47].The observation that dependence was associated with PTSD rather than trauma exposure is consistent with other studies [10]. Tobacco use may be seen as a form of self-medication to ameliorate symptoms of poor mental health, particularly in the case of PTSD.

While acknowledging that it is not possible in a cross-sectional study to determine causation (with some evidence among non-conflict-affected populations also indicating that tobacco use may increase susceptibility to poor mental health through a neural pathway [10,48,49]), the findings suggest that interventions may usefully address measures to reduce levels of PTSD and depression and nicotine dependence, potentially yielding synergistic benefits (as evidenced with US military veterans and general populations [47,48,49,50]). However, it is also necessary to obtain further evidence on the causal pathways and temporal nature of the relationship between mental disorders and nicotine dependence among conflict-affected civilian populations.

## 5. Limitations

First, the small number of women smokers meant they could not be included in the study. Second, as noted above, the cross-sectional study design means it cannot prove causation or explain temporal relationships. Third, the sample size may have prevented associations being observed with nicotine dependence, for example of individual trauma exposure items or anxiety. Fourth, the questionnaire did not include items on starting smoking which can be a key influence on later nicotine dependence [45]. It also did not include items about on knowledge of the health risks of smoking.

## 6. Conclusions

This study provides new evidence on smoking patterns and associations with common mental disorders among conflict-affected civilian populations. The observed associations between nicotine dependence, PTSD and depression suggests that programmes aimed at reducing the health burden from PTSD and depression may possibly also have an indirect effect in reducing the burden of nicotine dependence and that joint programmes may prove particularly effective. Further research is required on patterns of tobacco use and the effectiveness of mental health programmes in reducing nicotine dependence among conflict-affected civilian populations.
